# Impaired Facial Affect Perception in Unaffected Children at Familial Risk for Panic Disorder

**DOI:** 10.1007/s10578-014-0513-z

**Published:** 2014-10-15

**Authors:** Cynthia Bilodeau, Jacques Bradwejn, Diana Koszycki

**Affiliations:** 1 University of Ottawa, 145 Jean-Jacques Lussier, Ottawa, ON K1N 6N5 Canada; 2 l’Institut de recherche de l’Hôpital Montfort, Ottawa, ON Canada; 3 Present Address: St. Paul University, Ottawa, ON Canada

**Keywords:** Facial affect recognition, Emotion processing, Anxiety, Panic disorder, Children, Genetics, Endophenotype

## Abstract

Recent studies suggest that impaired processing of facial affect has a familial component and may reflect a marker of liability to psychopathology. This study investigated whether facial affect processing is impaired in offspring with parental panic disorder (PD). Psychiatrically healthy children with parental PD (n = 51) and age and sex matched control children with no parental psychopathology (n = 51) completed a standard facial recognition task. High-risk children made more errors recognizing fearful faces than controls and misattributed fear and angry facial affect as surprised. High-risk females also made more errors recognizing sad faces compared to low risk females and misattributed sadness as fear. No difference emerged for self-rated anxiety while viewing facial expressions. However, self-rated anxiety correlated moderately with misrecognition of fearful facial affect in high-risk children. Overall, our data suggest that the ability to correctly recognize negative facial emotions is impaired in children with parental PD. Further research is needed to confirm if these deficits represent a trait marker of liability for PD and elucidate the contribution of genetic and family environmental influences.

## Introduction

The ability to accurately decode facial emotions is important for adaptive social behaviors, emotional development, and well-being [1, 2]. Recognition of facial expressions begins in infancy and by middle childhood humans are capable of recognizing six basic facial expressions of emotion: anger, disgust, fear, happiness, sadness, and surprise [2, 3]. Developmental studies indicate that accuracy and speed in recognizing facial affect improve from childhood through early adulthood, plausibly due to maturation of brain structures associated with facial emotion processing and social experiential factors [3–5]. Individual differences in the ability to detect facial emotions accurately have been documented, especially for complex emotions such as fear and anger. Twin studies suggest that genetic and environmental influences likely account for individual differences in processing facial affect [6–8]. Although the role of specific genes and mechanisms are largely unknown, available molecular genetic data has linked facial affect processing with genes encoding neurotransmitter receptors, such catecholamine and serotonin receptors [9–13].

Impaired facial emotion processing has been observed in children and adolescents with a wide range of psychological problems including anxiety disorders [14, 15]. Studies using facial identification tasks report that anxiety disordered youth are less accurate in recognizing facial expressions than non-anxious controls. Specifically, anxious youth make more errors in recognizing negative valence faces [16–20] or mislabel positive or neutral emotions [16, 21]. Similarly, children with high trait anxiety have been found to make more errors recognizing negative valence faces than children with low trait anxiety [22]. Neuroimaging findings suggest that these deficits may be attributed to dysregulation of corticolimbic brain circuits [23–27], which are most often associated with the evaluation of facial expressions [28, 29]. Other studies, however, report that affect identification sensitivity is intact and unimpaired in anxious youth [30–35]. Inconsistent findings are likely attributed to differences in study population, sample size, medication use, and test design including range and intensity of facial stimuli used and exposure duration.

Little is currently known about whether impaired facial affect processing is a premorbid trait marker of anxiety disorder risk or a consequence of anxiety. Preliminary research with individuals at genetic risk for diverse psychiatric disorders suggests these deficits reflect a marker of liability to psychopathology. For example, unaffected first-degree relatives of probands with bipolar disorder [36], schizophrenia [37], and depression [38] have been found to exhibit deficits in labelling facial expressions compared to low-risk controls. Research on facial affect processing in relatives of probands with anxiety disorders is sparse. Pine et al. [39] investigated sensitivity and attention allocation to face photographs in offspring with and without parental panic disorder (PD) and found high-risk children to report more fear and longer latency to report fear during presentations of negative valence facial expressions than low-risk controls. However, as roughly half of the high-risk children had an anxiety disorder, it is unclear to what extent this sensitivity reflects a state or trait marker of anxiety. Nevertheless, this study does suggest that facial affect processing might be a promising mechanism for understanding the reported link between parental PD and elevated risk for the disorder in offspring [40].

The aim of the present study was to further explore facial affect processing in offspring with parental PD. We investigated psychiatrically healthy children in order to avoid the confounding effects of child psychopathology on outcome. Based on findings that patients with PD exhibit deficits in recognizing negative valence facial affect [41–43], especially threat-related expressions (i.e., anger and fear) which are postulated to signal threat in the environment via activation of the amygdala [29], we examined whether high-risk offspring would also exhibit deficits in recognizing threat-related emotions relative to low-risk controls. Further, based on findings by Pine and colleagues [39], we determined whether high-risk offspring would also report higher levels of subjective anxiety while viewing negative facial expressions of emotions than the control children. As sex can influence the accuracy of emotion recognition [3] and is a risk factor for PD [44], we also explored the interaction between risk group and sex on response to the facial recognition task. Additionally, the study controlled for the effect of anxious traits as they too may moderate response to facial affect processing tasks [22, 45].

## Materials and methods

### Participants

Facial recognition data were collected from children who participated in two separate studies on biological and psychological risk factors predisposing to anxiety. Children with and without parental PD were recruited through advertisements in local newspapers and the Internet and through posters in hospitals, family medicine centers and universities. To be eligible for the studies families had to have one or more biological child between the ages of 7–18 years with no documented history of psychiatric disorders. Children were excluded if they had a history of brain injury, clinically significant and/or unstable medical conditions, or used medications with peripheral or central nervous system effects. High-risk (HR) families had to have a parent with a current or past history of primary PD with or without agoraphobia (PD ± AG). For low-risk (LF) families neither parent could have a history of psychopathology. The institutional review boards approved the studies (REB #2008006 and #H- 09-09-08**)** and written informed consent was obtained from the child’s legal guardian as well as the child’s assent. Offspring 16 years and over provided their own consent. Families were compensated for their participation in the study.

### Assessment Procedures

Parents who expressed an interest in the research completed an initial telephone pre-screen with a research assistant who explained the purpose of the study and obtained information about the psychiatric status of both biological parents, as well as history of psychiatric symptoms, medical illness and medication use in their offspring. If the family was potentially eligible, a second telephone pre-screen was scheduled to confirm the diagnostic status of the parent. The DSM-IV based structured clinical interview (SCID) [46] was used to assess current or lifetime diagnosis of primary PD ± AG in the parent(s) of HR offspring and the absence of psychopathology in both parents of LR offspring. A registered psychologist conducted interviews with the affected parent and trained research assistants conducted interviews with the control parents.

Potentially eligible children were invited to the research laboratory for a face-to-face screen interview to confirm the absence of a current or past history of any Axis I disorder and other eligibility criteria. The child version of the SCID [47] was used to evaluate children who participated in study 1 and the child version of the Anxiety Disorders Interview [48] was used for children who participated in study 2. Interviewers were clinicians and trained research assistants who were unaware of parental diagnosis. Eligible offspring completed self-report questionnaires and returned to the laboratory for a second visit to complete the facial recognition task and other study procedures. Six children (one HR and five LR) were excluded from the study following the face-to-face assessment and three children (two HR and one LR) who were eligible to participate did not return for the second visit.

### Measures

#### The Pictures of Facial Affect (POFA) [1]

The POFA was used for facial emotions identification. The POFA includes 110 black and white photographs of 8 females and 6 males expressing either an emotion (happiness, sadness, fear, anger, disgust, or surprise) or a neutral facial expression. In the present study, participants were presented with pictures displaying 5 emotions (happiness, sadness, anger, fear, and surprise) or a neutral expression. We did not include disgust because it is the least accurately identified facial affect in children, with rates of accuracy averaging between 30 and 40 % [3]. There were 36 trials divided into 6 blocks of 6 pictures each (3 males, 3 females). Each block included pictures of all 5 facial affect and a neutral expression. Pictures were chosen at random and the presentation order was counterbalanced for each block. Pictures were presented sequentially on a computer screen using PowerPoint presentation. Children were provided with a list of all possible answers and after each picture had been displayed for 10 s, they were instructed to enter the correct choice by pressing a key on the computer keyboard. In the first 5 blocks children labelled the facial expressions. On the 6th block faces were presented in the same way as the others, however, participants were instructed to rate how anxious they felt while viewing each picture on a scale of 1 (not at all) to 5 (extremely).

#### State-Trait Anxiety Inventory for Children-Trait (STAIC-T) [49]

The STAIC-T is a well established self-report measure of trait anxiety in children. The scale is composed of 20-items rated on a 3-point scale, with higher scores indicating higher levels of trait anxiety. The STAIC-T correlates with childhood measures of anxiety and discriminates between children having high and low tendency to experience anxious states [50]. Retest reliability values range from 0.65 to 0.71 and the scale has satisfactory internal consistency, with an alpha coefficient of 0.80 [49]. In the current sample Cronbach’s alpha was 0.90.

#### Children Anxiety Sensitivity Index (CASI) [51]

The CASI is a self-report measure that assesses beliefs children have about the negative consequences of anxiety. It includes 18 items that are rated on a 3-point Likert scale. The CASI correlates with childhood measures of fear and anxiety [52, 53], discriminates between children with and without anxiety disorders [53], and predicts onset of spontaneous panic [54]. Psychometric evaluation of the CASI in children ages 6–17 years reveal internal consistency estimates of 0.87 and test–retest reliability estimates of 0.76 and 0.79 for nonclinical and clinical samples, respectively [51]. In the current sample Cronbach’s alpha was 0.83.

#### Child Self Report of Current Inhibition (CSRCI) [55]

The CSRCI is a 30-item self-report measure of childhood behavioral inhibition (BI) and is written in a language appropriate for children as young as seven years. The scale assesses behaviours related to inhibition including separation anxiety, social withdrawal, fears, and illness complaints, which are rated on a 5-point Likert scale. The content of the items on the CSRI directly parallel those of its adult equivalent, the Retrospective Childhood Inhibition Scale [55], from which the CSRI was derived. Although published data on the psychometric properties of the CSRI are currently unavailable, research on the RCIS indicates high internal consistency (αs range from 0.79 to 0.91). In the current sample Cronbach’s alpha was 0.80.

### Data Analysis

Data were analyzed with SPSS Version 21.0. Due to a problem with the computer keyboard data from 2.9 % of trials was missing. Little’s Missing Completely at Random (MCAR) test revealed no systematic pattern of missing data across the trials. Missing data for the number of errors committed and VAS anxiety ratings were imputed with the Expectation Maximization procedure. This imputation procedure is an acceptable practice for dealing with data missing completely at random. All available data were used for analysis of error patterns.

Mixed model analysis was used to determine the association between risk group and the number of errors committed in identifying faces, error patterns, and self-rated anxiety while viewing facial stimuli. The risk group × sex interaction was evaluated in the model and significant interactions resulted in subsequent stratified analysis. Family was included as a random effect in the model because some families included more than one child. A priori covariates included the STAIC-T, CASI and CSRCI. Age and ethnicity were also included as covariates in the model due to the wide age range of our sample and unexpected group difference in the proportion of Caucasian versus non-Caucasian children. Mean errors in recognizing happy, sad, surprised, mad, and neutral faces and VAS anxiety ratings while viewing happy, neutral, mad, and scared faces were skewed and analysis was performed on both untransformed and logarithmically transformed data. However, as results were comparable for both analyses untransformed results are reported herein. Effect sizes (Cohen’s d) and the 95 % confidence intervals (95 % CI) around the effect sizes were calculated to examine the magnitude of risk group differences. Finally, Pearson’s correlations were computed to assess the relationship between covariates and dependent measures and between errors in decoding facial affect and self-rated anxiety. Significance was set at 0.05 for all analyses. Due to the preliminary nature of this research and relatively small sample size we did not control for multiple tests.

## Results

### Sample Characteristics

Facial recognition data were available for 51 HR children from 38 families with parental PD. The affected parent was the mother in 71 % of families and in one family both parents had the disorder. The mean age of PD onset was 28.87 ± 10.5 years and 31 % of parents were in remission. HR children comprised 28 females and 23 males with a mean age of 10.9 ± 2.9 years. Forty-seven children were Caucasian (92 %), one was Asian (2 %), and three were mixed race (6 %). The 51 LR offspring derived from 43 families were matched for age and gender. Thirty-five were Caucasian (69 %), five were Asian (10 %), six were black (12 %), one was Hispanic (2 %), and four were mixed race (8 %). Child ethnicity differed between risk groups, with the HR group including a smaller percentage of non-Caucasian children than the LR group (Chi square = 6.47, *df* = 1, *p* = 0.012). Mean (±SD) scores for anxiety-related traits for high and low risk children were, respectively, 1.84 ± 0.4 and 1.81 ± 0.4 for the CSRCI, 26.47 ± 9.5 and 26.18 ± 5.3 for the CASI, and 30.88 ± 7.9 and 29.92 ± 7.0 for the STAIC-T. Differences between groups on mean anxiety-related trait scores were not statistically significant (*p*s ≥ 0.05). Correlations between covariates and dependent measures for the total sample are shown in Table 1.

**Table 1:** Correlations between covariates and total number of errors committed and anxiety ratings while viewing facial affect for the total sample

Dependent measures	Age	STAIC-T	CASI	CSRCI	Ethnicity of child
Number of errors committed					
Angry faces	−0.03	−0.14	−0.06	0.06	−0.03
Surprised faces	−0.16	0.05	0.11	0.14	−0.17
Neutral faces	−0.24*	0.10	0.08	0.08	−0.08
Sad faces	0.01	−0.01	0.01	0.11	0.12
Scared faces	−0.19	−0.12	−0.07	−0.03	−0.07
Happy faces	0.05	0.11	0.02	0.16	0.06
Anxiety while viewing facial affect					
Angry faces	−0.18	0.05	0.01	0.21*	−0.06
Surprised faces	0.08	0.17	0.09	0.2*	0.03
Neutral faces	−0.17	0.22*	0.14	0.33**	0.12
Sad faces	0.01	0.18	0.17	0.16	0.002
Scared faces	−0.22*	−0.10	0.02	−0.03	0.01
Happy faces	−0.06	0.14	0.15	0.27**	0.03

*STAIC*-*T* State-Trait Anxiety Inventory for Children-Trait, *CASI* Children Anxiety Sensitivity Index, *CSRCI* Child Self Report of Current Inhibition

* *p* < 0.05; ** *p* < 0.01

### Emotion Recognition and Processing

Mean errors in facial affect recognition are shown in Table 2. After controlling for age, ethnicity and anxiety-related traits, the analysis revealed a significant effect for risk group in errors committed identifying fearful affect, with HR children committing more errors than LR controls [F(1, 93) = 5.84, *p* = 0.018, Cohen’s d = 0.43 (95 % CI 0.04–0.82)]. Analysis of error patterns showed that HR children were more likely than LR controls to mislabel fearful facial affect as surprised [mean number fearful affect labelled as surprised: 1.90 ± 1.4 versus 1.28 ± 1.2 for HR and LR groups, respectively, F(1, 88) = 5.14, *p* = 0.026, Cohen’s d = 0.47 (95 % CI 0.08–0.87)]. A significant group × sex interaction [F(2, 93) = 4.35, *p* = 0.016] emerged for errors committed identifying sad affect, with HR females committing more errors than LR females [mean (±SD) error score: 1.39 ± 1.0 versus 0.60 ± 0.8, *p* = 0.002, Cohen’s d = 0.89 (95 % CI 0.32–1.42)]. No differences between high and low risk males were noted. Analysis of error patterns showed that HR females were more likely than LR females to mislabel sad affect as fear [mean (SD) number sad affect labelled as fear 0.48 ± 0.7 versus 0.12 ± 0.3, F(1, 44) = 5.30, *p* = 0.03, Cohen’s d = 0.70 (95 % CI 0.16–1.24)].

**Table 2:** Mean (±SD) errors committed identifying facial affect

Facial affect	High risk children	Low risk children	Cohen’s d (95 % CI)
Angry	0.97 ± 0.69	0.84 ± 0.78	0.11 (−0.28 to 0.50)
Surprised	0.47 ± 0.70	0.46 ± 0.65	0.01 (−0.37 to 0.40)
Neutral	1.03 ± 1.54	0.76 ± 0.81	0.22 (−0.17 to 0.61)
Sad	1.22 ± 1.02	0.95 ± 1.02	0.26 (−0.12 to 0.65)
Fearful	2.15 ± 1.47	1.57 ± 1.22	0.43 (0.04 to 0.82)
Happy	0.25 ± 0.52	0.24 ± 0.51	0.02 (−0.37 to 0.40)

Values are unadjusted means. *CI* confidence intervals

The number of errors committed identifying neutral, happy, surprised, and angry faces was comparable for high- and low-risk children (*p*s ranged from 0.28 to 0.84 for risk group main effects and from 0.15 to 0.75 for risk group × sex interactions). When the error pattern for these faces was examined, HR children were more likely than LR controls to mislabel angry faces as surprised (mean (SD) number of angry faces labelled as surprised 0.57 ± 0.5 versus 0.33 ± 0.5 [F(1, 86) = 8.25, *p* = 0.01, Cohen’s d = 0.49 (95 % CI 0.10–0.89)].

Mean anxiety ratings while viewing each affect are shown in Table 3. A significant group × sex interaction emerged for fear while viewing angry faces [F(2, 93) = 3.79, *p* = 0.03], however none of the pairwise comparisons were statistically significant. No effect of group or group × sex interaction was detected for the other anxiety ratings (*p*s ranged from 0.17 to 0.90 for risk group main effects and from 0.16 to 0.96 for risk group × sex interactions). Correlation analysis revealed that among HR children, a significant positive association emerged between anxiety ratings and the number of errors committed identifying fearful (r = 0.37, *p* = 0.008) and neutral (r = 0.30, *p* = 0.03) affect. Among LR children, a significant positive correlation emerged between anxiety ratings and the number of errors committed identifying neutral face (r = 0.34, *p* = 0.02). Differences between correlation coefficients between high- and low-risk children were not statistically significant (*p*s ranged from 0.37 to 0.88).

**Table 3:** Mean (±SD) anxiety ratings while viewing facial affect by risk group

Facial affect	High risk children	Low risk children	Cohen’s d (95 % CI)
Angry	1.56 ± 0.89	1.46 ± 0.82	0.10 (−0.08 to 0.49)
Surprised	2.67 ± 1.28	2.78 ± 1.24	−0.15 (−0.54 to 0.23)
Neutral	1.94 ± 1.07	2.00 ± 1.00	−0.14 (−0.52 to 0.25)
Sad	2.46 ± 1.05	2.40 ± 1.17	0.02 (−0.37 to 0.41)
Fearful	1.34 ± 0.88	1.65 ± 1.15	−0.34 (−0.73 to 0.05)
Happy	1.90 ± 0.91	2.04 ± 1.10	−0.17 (−0.56 to 0.21)

Values are unadjusted means. *CI* confidence intervals

## Discussion

To our knowledge this is the first study to assess facial affect recognition in unaffected offspring with parental PD. The results indicate that HR children exhibit deficits in recognizing specific negative valence emotions, whereas recognition of positive and neutral affect appears to be intact. As predicted, HR children made more errors decoding fearful faces than LR controls. This finding is consistent with studies demonstrating that anxious youth [20] and adult patients with PD [41–43] exhibit deficits in recognizing threat-related facial expressions such as anger and fear. Further analysis indicated that HR children mislabeled angry and fearful faces as surprised, although the significance of this finding is unclear as surprised is an ambiguous facial expression that allows for either positive or negative interpretations [56]. However, recent research by Tottenham et al. [57] revealed that children and adolescents typically show a negative bias when viewing surprised faces compared to adults, and that confusion between angry, fearful, and surprised faces in youth is likely due to a negative interpretation of surprised faces rather than perceptual similarity. This would suggest that our HR children exhibited a negative bias when they mislabeled anger and fear as surprised, although we cannot exclude the possibility that these emotions were also misperceived as a positive valence affect.

Our data also showed a significant difference between PD risk groups in the capacity to discriminate sad faces based on gender. Specifically, HR females made more errors in identifying sad affect than LR females and misperceived sadness as fear. Adult PD patients have also been reported to have lower accuracy in recognizing sad affect and this pattern of error correlates with severity of depressive symptoms [41]. This finding is not surprising as many of the brain structures involved in the processing of sad and fearful facial affect overlap [58]. Individuals at risk for depression are faster at recognizing fearful faces than LR controls [59] and brain imaging studies have found youth at familial risk for depression to show greater amygdala activation in response to fearful facial expressions than LR controls [15]. Children with a history of major depression show perturbed encoding of fearful faces [60] and depressed girls exhibit a blunted amygdala response to fearful faces compared anxious and healthy control girls [23]. A blunted amygdala response to fearful faces has also been observed in medicated adults with PD and is thought to reflect a compensatory response to amygdala hyperreactivity to threat-related stimuli [61]. Considering that panic attacks, PD and depression are highly comorbid [62], it is possible that deficits in recognizing sad facial affect is a vulnerability marker of risk for depression in females with parental PD. This question merits further empirical research.

We could not confirm our hypothesis that HR children would also experience more subjective anxiety while viewing facial affects compared to control children. Although Pine et al. [39] reported that offspring at risk for PD experienced more fear while viewing evocative facial affect than LR offspring, their HR sample included symptomatic children which may have confounded findings. Unlike Pine et al.’s study we did not measure reaction time for rating facial affect and cannot exclude the possibility that our unaffected HR children would have exhibited a slower reaction time for rating negative valence emotions than controls. We also measured subjective anxiety in the final block of face presentations and differences between risk groups may have emerged if we obtained anxiety ratings during additional blocks of face presentations. Nevertheless, in contrast to LR children, HR children exhibited a moderate and significant positive correlation between self-report anxiety and errors recognizing fearful affect, suggesting that viewing this emotion evoked heightened emotional reactivity in these children.

The facial recognition task is purported to provide a window into the functioning of the amygdala [28, 39]. Dysregulation of the amygdala has been implicated in both the pathophysiology of PD [63] and impaired face-emotion processing in anxious and anxiety-prone individuals [16, 23–27, 64]. We are unaware of any published neuroimaging studies of facial affect recognition in unaffected offspring at familial risk for panic or other anxiety disorders. This type of research is needed to map brain networks involved in the processing of facial affect in these vulnerable individuals. Prospective neuroimaging studies are also needed to investigate whether face processing brain networks in unaffected at-risk offspring are stable or change over time due to maturation or other processes, such as exposure to negative life events which often precipitate onset of panic attacks [65, 66] or the emergence of subsyndromal symptoms of pathological anxiety. Such research holds promise to advance our understanding of the trajectory of PD risk and course of illness, and elucidate whether alterations in brain networks presumed to be involved in facial affect processing are a general trait marker of risk or secondary to anxiety states.

Overall, our data concur with reports that deficits in emotion recognition may have a familial component [36–38]. PD aggregates in families [40, 67] with heritability estimated at 40 % [67]. Although many genetic polymorphisms have been tested in association studies the genetic basis for PD is largely unknown [68]. A more recent focus in genetic research of PD has been to discover heritable trait markers of disorder vulnerability. Our preliminary data suggest that deficits in processing fearful and sad affect may be a potential trait marker, although more research is needed to replicate findings and establish if these deficits meet criteria for an endophenotype. From a genetic perspective, there is supportive evidence the serotonin transporter gene (5-HTT) may play a role in the processing of fearful facial affect. Young children with the short allele of the 5-HTT gene have been reported to be less accurate in recognizing fearful faces but not other facial affect than children with the long allele [69]. In a study of undergraduate students, short allele carriers of the 5-HTT gene were more accurate in recognizing fearful affect but less accurate in recognizing happy affect than long allele carriers [10]. The differential impact of the 5-HTT gene on recognizing fearful faces in children versus young adults possibly reflects developmental changes whereby deficits transition from poor identification to hypervigilance. Several imaging genetic studies have also shown that variation of the 5-HTT gene confers heightened amygdala reactivity to threatening facial emotions [70–72]. It would be worthwhile for future research to determine if the 5-HTT gene and other candidate genes associated with PD risk confer deficits in facial affect recognition in unaffected offspring with parental PD.

In addition to genetic influences it is important to consider the influence of family environment in shaping children’s emotion recognition skills. There is emerging evidence that parenting style and parent–child attachment can influence a child’s ability to accurately recognize and interpret emotional cues [73–75]. As parents with PD have been reported in some studies to have a rejecting and overprotective parenting style [76, 77], it is plausible that negative parenting mediates the relationship between parental PD and impaired facial affect recognition in offspring. Parental displays of negative emotions, especially anger and criticism, have also been associated with impaired processing of facial affect in children [11, 78, 79]. While no study to date has examined the impact of parental expressions of anxiety on children’s facial affect perception skills, parental modeling of anxious behaviour and cognitions has been reported to have discernable effects on children’s perception of threat [80, 81]. In addition to parental displays of emotions, parental masking of emotions can potentially influence how children learn to recognize emotional faces. Parents who believe emotions are dangerous tend to mask emotional expressions, perhaps in an attempt to shield their child from observing negative emotionality [82]. As patients with PD have negative beliefs about the consequences of anxiety, it is possible that some parents with the disorder suppress emotional expressions of fear in the presence of their children. Although the precise mechanisms are unknown, parental masking of fear could influence how offspring process threat-related facial stimuli. Considering that parents play a pivotal role in their child’s emotional socialization, additional research is needed to clarify the role of family environment on facial affect perception in children with parental PD.

While this study has significant strengths related to the recruitment of psychological healthy offspring of parents with SCID confirmed PD, limitations should be acknowledged. First, the sample size is relatively small and we may not have had sufficient power to detect other differences in facial affect recognition or anxiety ratings while viewing negative valence faces. Second, families were recruited via advertisement and therefore self-selected themselves into the study, limiting generalizability of findings. Third, this study is cross-sectional and we cannot confirm that deficits in facial recognition predict risk for onset of pathological anxiety. Longitudinal studies are needed to determine whether deficits are more common in HR offspring who eventually develop symptoms of anxiety versus those who do not. Fourth, while failure to identify facial expressions correctly is believed to serve as a risk marker for social maladjustment, we did not examine whether performance on the facial recognition task correlates with impaired social interactions. Given that onset of PD has been linked with interpersonal stressors [83] and that many anxiety disordered patients report relational difficulties [84], further work should investigate whether impaired performance on facial cognitive tasks in HR children correlates with problematic processing of social cues in real life social interactions. Fifth, the age range of our sample was relatively broad and it is possible that developmental differences in face-emotion processing influenced outcome. However, as the correlation coefficients between age and number of facial recognition errors were small, it is unlikely that age was a significant predictor of response to the facial recognition task. Another study limitation is that our HR group comprised predominately Caucasian children and high- and low-risk children were not matched on ethnicity. A more ethnically diverse sample may have yielded different results. Finally, we used standard pictures of facial affect of Caucasian posers. It is possible that low intensity emotional expressions, morphed facial expressions and faces from different ethnicities would reveal more subtle differences between risk groups.

## Summary

To summarize, the main finding of this study is that psychiatrically healthy children with parental PD exhibit deficits in recognizing negative valence facial emotions. Whether these deficits play a pathogenic role in the development of anxiety in at-risk children remains to be determined in prospective longitudinal research. Although our findings are preliminary and require replication in a larger sample, results could have implications for the development of preventive interventions. Recent work with autistic [85] and depressed [86] children suggests that facial emotion recognition training improves social cognition. Similar findings have been reported in adults with severe psychiatric disorders [87] and elevated levels of depressive symptoms [88]. Preventive interventions that incorporate strategies to reduce biased processing of facial signals that can lead to inappropriate social responses and interpersonal difficulties may also be of benefit to children at risk for panic and other anxiety disorders.
